# Diurnal Fluctuations of Verticality Perception – Lesser Precision Immediately after Waking up in the Morning

**DOI:** 10.3389/fneur.2015.00195

**Published:** 2015-09-02

**Authors:** Aline J. Schwarz, Dominik Straumann, Alexander A. Tarnutzer

**Affiliations:** ^1^Kantonsschule Wettingen, Wettingen, Switzerland; ^2^Department of Neurology, University Hospital Zurich, University of Zurich, Zurich, Switzerland

**Keywords:** vestibular, multisensory integration, perception, sleep, otolith organs

## Abstract

Internal estimates of direction of gravity are continuously updated by integrating vestibular, visual and proprioceptive input, and prior experience about upright position. Prolonged static roll-tilt biases perceived direction of gravity by adaptation of peripheral sensors and central structures. We hypothesized that in the morning after sleep, estimates of direction of gravity [assessed by the subjective visual vertical (SVV)] are less precise than in the evening because of adaptation to horizontal body position and lack of prior knowledge about upright position. Using a mobile SVV-measuring device, verticality perception was assessed in seven healthy human subjects on 7 days in the morning immediately after waking up and in the evening while sitting upright. Paired *t*-tests were applied to analyze diurnal changes in SVV trial-to-trial variability. Average SVV variability in the morning was significantly larger than in the evening (1.9 ± 0.6° vs. 0.9 ± 0.3°, *p* = 0.002). SVV accuracy was not significantly different (−1.2 ± 0.9° vs. −0.4 ± 0.4°, morning vs. evening, *p* = 0.058) and was within normal range (±2.3°) in all but one subject. A good night’s sleep has a profound effect on the brain’s ability to estimate direction of gravity. Resulting variability was significantly worse after waking up reaching values more than twice as large as in the evening while there was no significant impact on SVV accuracy. We hypothesize that lacking prior knowledge, adaptation of peripheral sensors, and lower levels of arousal and cerebral metabolism contribute to such impoverished estimates. Our observations have considerable clinical impact as they indicate an increased risk for falls and fall-related injuries in the morning.

## Introduction

For successful spatial orientation and navigation, a stable and upright body position relative to gravity is essential. Internal estimates of direction of gravity are continuously generated based on input originating from different sensory systems including the vestibular organs [containing both the otolith organs for detecting linear acceleration and the semicircular canals (SCCs) for measuring angular accelerations], proprioceptors located on the skin (for pressure monitoring) and in the joints (registering the relative position of individual parts of the limbs) and vision [see Ref. (1) for review]. Amongst these different systems, the otolith organs take a key position as they are the only sensors that directly detect linear acceleration and pull of gravity (2). Besides sensory input, also recent experience (“prior knowledge”) about earth-vertical position is taken into account when estimating direction of gravity (3, 4). According to Bayesian observer theory, the estimated direction of gravity is derived from the posterior probability distribution by combining sensory input and prior knowledge about earth-vertical in a statistically optimal fashion (5–7).

Internal estimates of direction of gravity, however, are not stable over time but fluctuate and drift over time both in upright (8) and roll-tilted positions (9–12). When changing whole-body roll position after prolonged static roll-tilt, also a bias of perceived direction of gravity toward the previous roll-tilted position – termed “post-tilt bias” is noted (12, 13). Both adaptations of the peripheral sensors and of central structures integrating graviceptive input have been proposed to explain such fluctuations and biases (12). By updating internal estimates of direction of gravity continuously, the brain is able to adapt quickly to changing environmental conditions. Such plasticity is essential to prevent falls due to instable body positions [as demonstrated by the correlation between risk of fall and postural instability on force posturography in elderly active people (14)] and allow interaction with external objects.

While frequent changes in head and trunk position during daytime will result in continuous updating of our internal estimates of direction of gravity, we remain in a lying position at night for several hours. Changes in body position also occur during sleep; however, the frequency and extent of such changes are much smaller than during daytime. Furthermore, there is no need to tightly monitor direction of gravity during sleep as we usually remain in a stable lying position. Based on few shifts in body position relative to gravity at night, lack of prior knowledge about upright and reduced brain activity during sleep and when waking up, we hypothesize that immediately after waking up, internal estimates of direction of gravity are not optimized yet (i.e., more variable). We propose the use of the subjective visual vertical (SVV) (15) to behaviorally quantify the precision (i.e., the degree of reproducibility as reflected by the trial-to-trial variability) of SVV adjustments (4) and predict reduced precision immediately after waking up in the morning compared to during later in the day. Clinically, this may be reflected in the first steps after standing up to be broad-based and ataxic, accompanied by a sense of gait imbalance, especially if the light is dim or kept off during this.

## Materials and Methods

In this study, seven healthy human subjects (five female and two male participants, aged 17.4 ± 1.9 years, average ±1 SD) were studied. Informed consent of all subjects was obtained after a full explanation of the experimental procedure. The protocol was accepted by the Cantonal Ethics Committee, Zurich and was in accordance with the ethical standards laid down in the 2013 Declaration of Helsinki for research involving human subjects.

### Experimental setting

**Figure 1:**
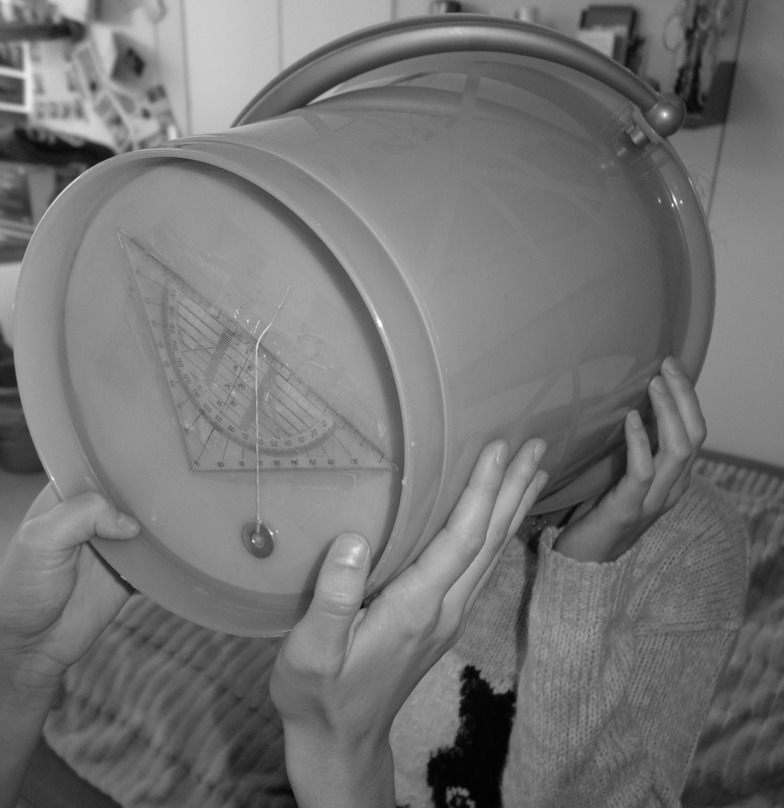
**Illustration of the mobile SVV-measuring device (“SVV bucket”) used in this study**. SVV adjustment errors were read out from a geometric triangle and a pendulum attached to the outside of the bottom of the bucket. Inside, at the bottom, a black line was drawn and the periphery of the bottom was covered to avoid light shining through the bottom of the bucket and provide external visual references.

All recordings were obtained at the participants’ homes by Aline J. Schwarz. Subjects were requested to sleep at least 6 h in the night before and not to consume alcohol or illicit drugs in the previous 24 h. Participants were seated upright at the side of their beds with the head straight-ahead in an upright position. SVV measurements were obtained with a mobile SVV measurement device (“SVV bucket”; Figure 1) inspired by SVV devices described previously (16). At the bottom of this bucket, a black line (6 cm long) was drawn. For each trial, the experimenter positioned the bucket in front of the participant and the participant rolled the bucket clockwise or counter-clockwise so that the line was parallel to perceived direction of gravity. A pendulum attached to a triangle was placed outside at the bottom of the bucket, allowing reading out the roll position of the line relative to gravity by the experimenter.

### Experimental paradigm

In each subject, SVV measurements were obtained in the morning between 5.45 a.m. and 10.20 a.m. immediately after waking up and in the afternoon/evening between 12 p.m. and 22.30 p.m. on seven non-consecutive days. Before the morning sessions, the experimenter woke up the participant and turned on the light. All recordings were obtained between 20th June and 16th August, 2014. In each session, six trials with different starting positions (±30°, ±20°, and ±10°) were completed. There was no time limit for individual adjustments.

### Statistical analysis

For both morning and afternoon/evening recording sessions and individual subjects, the trial-to-trial variability (equivalent to 1 SD) was determined. Statistical analysis was based on paired *t*-tests (ttest2.m, Matlab, The MathWorks, Nantuck, USA) and non-parametric analysis of variance (Kruskal–Wallis ANOVA) with Tukey–Kramer correction for multiple tests. The level of significance (*p*) was set to 0.05.

## Results

**Figure 2:**
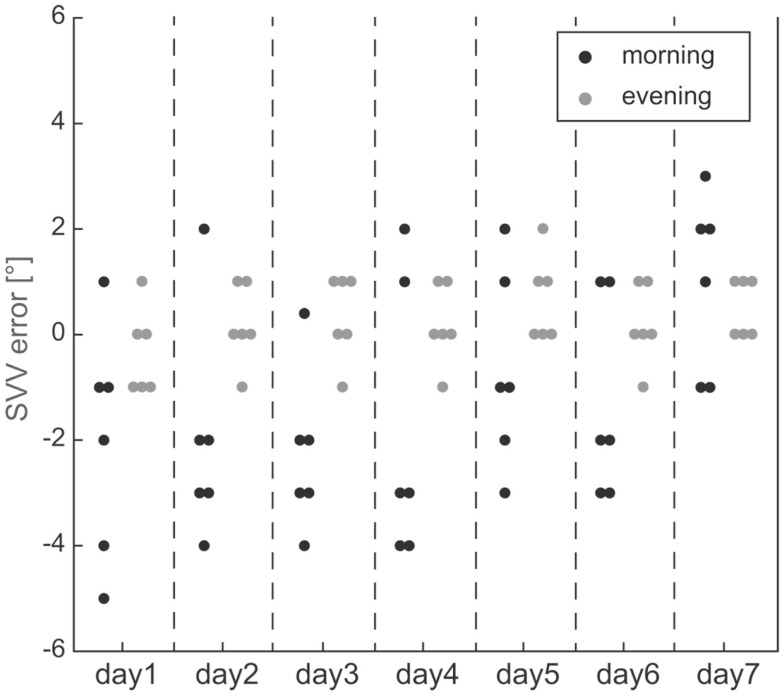
**Illustrative example of SVV adjustments in a single subject (#7)**. Individual adjustment errors are indicated by dots. The comparison between morning (black dots) and evening (gray dots) recording sessions (showing recordings from all 7 days) indicated overall larger trial-to-trial variability for all morning sessions.

**Figure 3:**
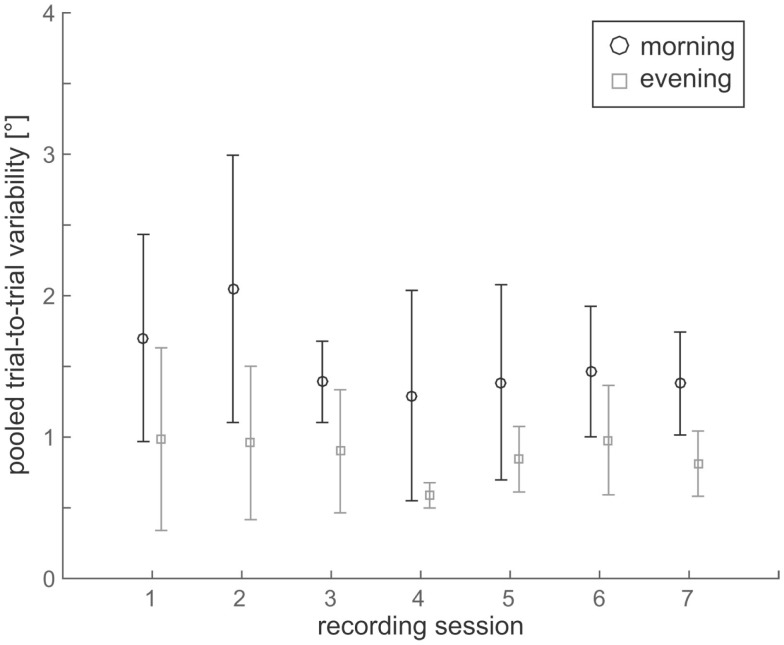
**Comparison of trial-to-trial variability values over the 7 days of data acquisition both in the morning (black circles) and in the evening (gray squares) for all seven subjects**. Statistical analysis showed no significant differences in variability between the seven recording sessions – this was true for both morning and evening sessions.

**Figure 4:**
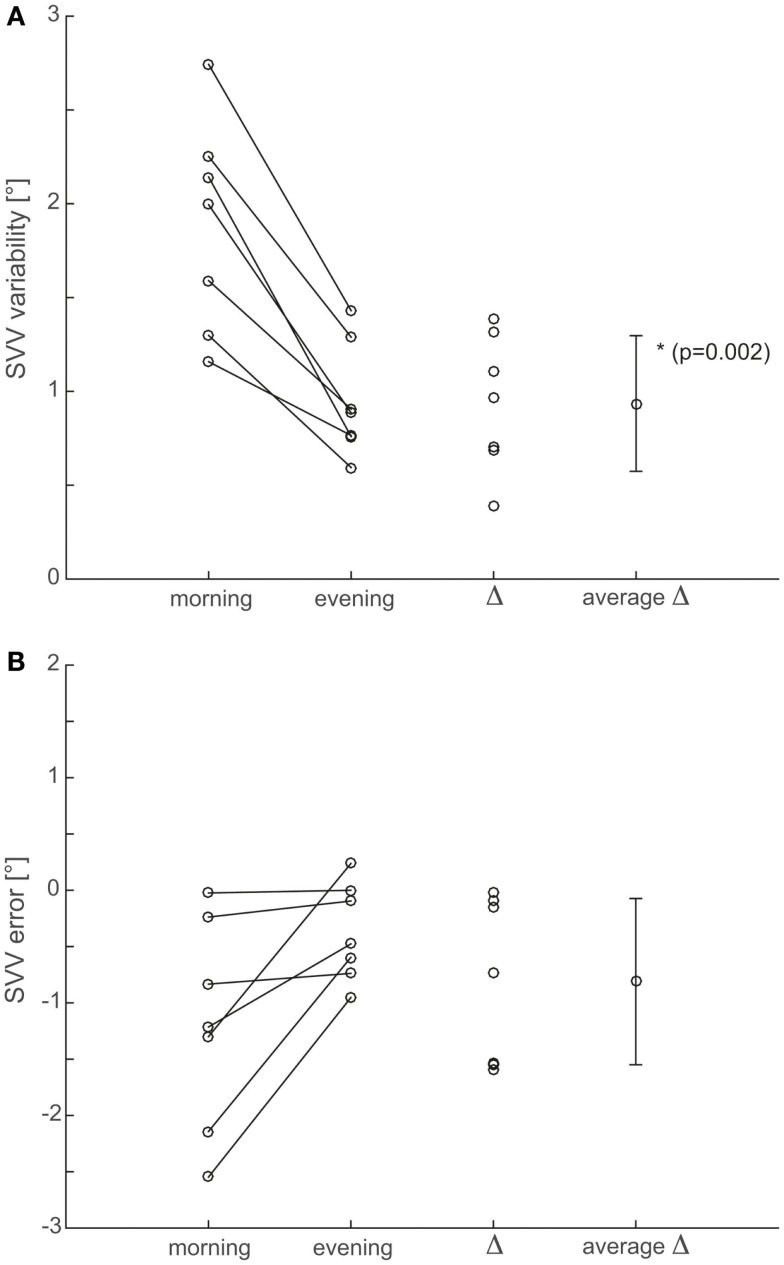
**Summary of both precision (A) and accuracy (B) of SVV adjustments**. To improve statistical power, data points from all seven recording sessions were pooled after confirming that there were no statistically significant (*p* > 0.05) differences between single sessions. For both precision (i.e., trial-to-trial variability) and accuracy (i.e., adjustment errors), individual average values from recordings in the morning (first column from left) and in the evening (second column from left) are compared for all seven subjects. Furthermore, individual differences (Δ, third row) and average (±1 SD) differences (average Δ, fourth row from left) are shown.

Individual trial-to-trial variability values were determined in all participants. Overall, we noted increased variability in all seven subjects in the morning sessions compared to the evening sessions (see raw single subject data in Figure 2). In order to increase sample size and statistical power, adjustments from the seven sessions were pooled (done separately for morning and evening sessions) before the average adjustment error and the trial-to-trial variability were determined. This was based on statistical analysis (Kruskal–Wallis ANOVA) previously obtained, demonstrating no significant differences (*p* > 0.05) between the seven morning sessions and between the seven evening sessions (see Figure 3). With such pooled data, we found significantly (*p* = 0.002, paired *t*-test) increased trial-to-trial variability in the morning session compared to the evening session (1.9 ± 0.6° vs. 0.9 ± 0.3°, average of individual variability values ±1 SD), as illustrated in Figure 4A.

For the analysis of trial-to-trial variability, we pooled individual adjustments from the seven recording sessions for the analysis of adjustment errors as we did not find significant (*p* > 0.05, Kruskal–Wallis ANOVA) differences between recording sessions. Individual average adjustment errors were either smaller (*n* = 6) or unchanged (*n* = 1) in the evening sessions compared to the morning sessions (−0.4 ± 0.4° vs. −1.2 ± 0.9°; overall average ±1 SD). However, these reductions in adjustment errors were not significant (*p* = 0.058, paired *t*-test) and with the exception of one individual subject, individual average adjustment errors from the morning sessions were within the range of normal (i.e., ±2.3°) as shown in Figure 4B.

Previously a post-tilt bias with fast (time constant approximately 70 s) exponential decay has been observed when returning back upright after 5 min of static roll-tilt (12). Possibly, re-adaptation of verticality estimates may have contributed to increased trial-to-trial variability in the morning sessions immediately after waking up. We, therefore, compared absolute adjustment errors (pooled data from all subjects) over time (i.e., over the series of six adjustments). While we found a trend toward somewhat smaller adjustment errors on an average in the morning sessions (decreasing from 2.2° to 1.5°), the differences among the six adjustments were not statistically significant (Kruskal–Wallis ANOVA with multiple comparisons, *p* > 0.05).

We also interviewed all participants about their percept of imbalance of stance and gait when getting up in the morning. Interestingly, only one out of seven subjects actually felt unstable in the morning after getting up, while in the other six subjects, the increased trial-to-trial variability in estimated direction of gravity was not reflected in subjective impairments of balance.

## Discussion

Internal estimates of direction of gravity are continuously updated by the brain based on an optimal integration of multisensory input and prior knowledge in order to allow accurate and precise orientation and navigation in space (1, 5, 6). While internal estimates of earth-vertical are very reliable for common conditions, such as body-upright position during daily activities, we predicted inferior performance after prolonged horizontal body positions, such as experienced during sleep. This hypothesis is confirmed by our experimental data with trial-to-trial variability of SVV estimates being more than twice as large in the morning immediately after waking and sitting up compared to the evening in healthy human subjects. At the same time, overall adjustment errors were within the range of normal as previously reported by others [±2.3°(16)] using a similar SVV bucket device both for morning and evening measurements. Taken together, our observations suggest a diurnal dissociation between SVV variability and SVV errors.

### Possible explanations for lower SVV precision in the morning

Increases in trial-to-trial variability reflect an impaired (or more noisy) “calibration” of the neuronal networks that generate internal estimates of direction of gravity and may emerge from different sources, including adaptation of peripheral sensors, reduced central sensory integration, and lacking recent experience about upright position when waking up.

#### The Contribution of Lower Levels of Arousal Immediately After Waking Up

The cerebellum plays an important role in many adaptive mechanisms. This includes adaptation of the otolith-ocular reflex and loss of cerebellar function, therefore, resulting in downbeat nystagmus that is typically worst in the morning (17) and increased trial-to-trial SVV variability (18). In accordance with this observation, these patients also report a peak in gait unsteadiness in the morning. It is conceivable that this mechanism also plays a role in subjects with preserved cerebellar function, however, with a much shorter time course.

Central integration of graviceptive input (e.g., at the level of the vestibulo-cerebellum or the multisensory vestibular cortex) may be impaired immediately after waking up due to lower levels of arousal [Arousal Hypothesis (19)] and decreased cerebral metabolism (20) reflected in a general slowing down of cognitive processes independently of the task and its level of difficulty. This phenomenon of decreased performance and/or disorientation occurring immediately after awakening from sleep compared to the pre-sleep status is referred to as “sleep inertia” and depends on prior sleep duration and sleep deprivation, sleep stage prior to awakening and time-of-day of awakening (21). Sudden awakening from sleep – as it was the case in our participants for the morning sessions – has been linked to decreased performance for many tasks including coordination (22). While most studies have addressed sleep inertia after short naps, it also occurs after a normal 8-h sleep period with afterward gradual increase in alertness and gradual decrease in reaction time (23). The time course of sleep inertia is still debated, with duration reported in various studies ranging between 1 min and 4 h, but rarely exceeds 30 min in the absence of major sleep deprivation (21). Sleep inertia may have a stronger effect on reaction time than on accuracy (21). Considering the short latency (in the range of few minutes) of SVV testing after waking up, it is very likely that measurements fell within the period of decreased cognitive performance due to sleep inertia and therefore also resulted in modifications of the effectiveness of sensory systems and central integration of graviceptive sensory input. Noteworthy, effects of sleep inertia on verticality perception have not been systematically studied in the past. However, considering the broad and very general effect of reduced levels of arousal on (especially higher) cognitive functions (21), an impact on internal estimates of direction of gravity seems very likely. For postural control – a task that also requires accurate and precise estimates of direction of gravity – varying performance depending on the level of vigilance and sleepiness (24–27) and an influence of the time of the day were described (28–31). Specifically, improvements in postural control were reported for evening sessions (measurements at 6 p.m.) compared to morning sessions (measurements at 6 a.m.) and were linked to increases in body temperature and sleepiness/vigilance levels improvement throughout the day (31). In this context, it was also proposed that cerebellar regulation improves throughout the day in order to regulate postural sway more efficiently (31).

Noteworthy, recording times in the morning and the evening were not strictly defined but were adapted to usual sleeping and working habits of the participants. Diurnal fluctuations – as described for many cognitive tasks including in the performance of postural control (31) – were, therefore, not controlled for and may have affected measurements in individual sessions and subjects differently.

#### The Role of Peripheral Sensory Adaptation After Sleep

Sensory adaptation is known to occur for almost all sensory systems [see, for example, Ref. (32, 33)] and was linked to changes in perceived direction of gravity after prolonged static (roll-titled) body positions as well (12, 13). After prolonged body horizontal position, important sensory systems for estimating direction of vertical, including skin proprioceptors and the hair cells within the otolith organs, have adapted to the most recent (horizontal) position. During the process of “re-calibration” after standing up, verticality estimates likely fluctuate over time. Besides increased trial-to-trial variability, such behavior may also result in drift if fluctuations are preferentially into one direction (instead of being random). Our data, however, speaks against a relevant role of drifting verticality estimates immediately after waking/standing up as over the series of six SVV adjustments obtained here, we did not observe significant changes in the absolute error.

Input from the different vestibular sensors converges at the level of the vestibular nuclei. Noteworthy, in non-human primates, vestibular neurons that receive otolith input only, i.e., that are focusing on head roll-tilt relative to gravity, prolonged head roll resulted in little or no adaptational changes (34). This suggests that the increase in trial-to-trial variability of perceived vertical observed in our study subjects rather originates from other (higher cortical) neuronal networks. Such a dissociation may emphasize the potential bimodal nature of internal and conscious perception.

#### Lacking Recent Experience About Upright Position After Sleep

According to Bayesian optimal observer theory, recent experience is important for improving internal estimates of direction of gravity (3, 5, 6). Immediately after sleep, however, there will be lack of prior knowledge about body position relative to gravity, followed by more noisy estimates due to the short duration of past experience (after waking up). This likely makes estimates more variable and more prone to biases introduced by imperfections of the sensory input signals integrated.

Taken together, the significant increases in SVV trial-to-trial variability immediately after waking/standing up are likely linked to several causes. This includes both residual sleep inertia immediately after waking up and diurnal fluctuations in task performance for many tasks, including postural stability – and likely also verticality perception – sensory re-adaptation and lacking prior knowledge. To our knowledge, there is no published research on internal estimates of direction of gravity immediately after getting up in the morning. Noteworthy, studies regarding diurnal fluctuations in postural control were designed differently [e.g., Ref. (31)] as subjects were already 1 h awake before morning sessions, excluding any effect related to prolonged static whole-body roll.

### The impact of impaired verticality perception when waking/standing up

Such diurnal fluctuations in the precision of internal estimates of direction of gravity have also important clinical implications as they may worsen postural stability and gait in the morning. While such fluctuations are physiological and will remain unnoticed by most healthy human subjects, they may add up with other conditions that impair balance and gait, such as orthostatic dysregulation, polyneuropathy, leg paresis, or impaired vision, resulting in increased rates of falls and fall-related injuries in these patients. Optimizing vision (i.e., turning on the lights) and probably getting up in steps (with a resting period sitting at the edge of the bed) may constitute helpful approaches to minimize falls and should be implemented consequently.

In summary, we confirm that prolonged horizontal body position, such as after a good night’s bed rest (>6 h) significantly impairs the brain’s ability to estimate direction of gravity immediately after waking and getting up. As outlined above, this has immediate clinical implications as both healthy human subjects and even more patients with gait and balance impairment are at increased risk for falls and fall-related injuries. Most likely, inferior performance in SVV precision in the morning immediately after waking/standing up is multifactorial, with ongoing sleep inertia, diurnal fluctuations in cognitive performance, lack of prior knowledge, and sensory re-adaptation as potential contributors.

## Author’s Contributions

AS, DS, and AT conceived of the study and formulated the study hypotheses. AS performed the experiments. AT performed the statistical analysis. AT drafted the manuscript. AS and DS participated in the study design, its coordination, and interpretation of the results. All authors read and approved the final manuscript.

## Conflict of Interest Statement

The authors report no conflict of interest. The funding sources had no involvement in the study design, the collection, analysis and interpretation of the data, the writing of the report, or in the decision to submit the paper for publication.
